# Whole blood circular RNA hsa_circ_0002171 serves as a potential diagnostic biomarker for human adenovirus pneumonia in children

**DOI:** 10.1590/1414-431X2022e12347

**Published:** 2022-11-04

**Authors:** Diyuan Yang, Ke Sun, Feng Huang, Huifeng Fan, Tingting Shi, Xinxin Chen, Gen Lu

**Affiliations:** 1 The First Affiliated Hospital, Jinan University, Guangzhou, Guangdong, China; 2 Department of Respiration, Guangzhou Women and Children's Medical Center, Guangzhou Medical University, Guangzhou, Guangdong, China; 3 Department of Anesthesiology, Guangdong Provincial People's Hospital, Guangdong Academy of Medical Sciences, Guangzhou, Guangdong, China; 4 The Second Affiliated Hospital, Guangzhou Medical University, Guangzhou, Guangdong, China

**Keywords:** Children, Circular RNA, Adenovirus pneumonia, Regulatory roles, Diagnostic biomarker

## Abstract

Severe pneumonia related to human adenoviruses (HAdVs) has a high lethality rate in children and its early diagnosis and treatment remain a major challenge. Circular RNAs (circRNAs) are novel long noncoding RNAs that play important roles in gene regulation and disease pathogenesis. To investigate the roles of circRNAs in HAdV pneumonia, we analyzed the circRNA profiles of healthy children and children with HAdV pneumonia, including both mild and severe cases, and identified 139 significantly upregulated circRNAs in children with HAdV pneumonia *vs* healthy controls and 18 significantly upregulated circRNAs in children with severe HAdV pneumonia *vs* mild HAdV pneumonia. In particular, hsa_circ_0002171 was differentially expressed in both groups and might thus be useful as a diagnostic biomarker of HAdV pneumonia and severe HAdV pneumonia. To identify the underlying mechanisms of circRNAs in HAdV pneumonia, we analyzed the transcriptome of children with HAdV pneumonia and established a circRNA-mRNA regulatory network. Enrichment analysis of differentially expressed target mRNAs demonstrated that the differentially expressed genes between healthy controls and HAdV pneumonia patients were mainly involved in RNA splicing while the differentially expressed genes between children with mild and severe HAdV pneumonia were mainly involved in regulating lymphocyte activation. Receiver operating characteristic (ROC) curve analysis suggested that hsa_circ_0002171 had a significant value in the diagnosis of HAdV pneumonia and of severe HAdV pneumonia. Taken together, the circRNA expression profile was altered in children with HAdV pneumonia. These results demonstrated that hsa_circ_0002171 is a potential diagnostic biomarker of HAdV pneumonia.

## Introduction

Human adenoviruses (HAdVs) belong to the *Adenoviridae* family and are nonenveloped double-stranded DNA viruses. As major causes of acute respiratory tract illness in children, HAdVs account for 3.8-11% of pneumonias (1-3). HAdVs have the strongest correlation with severe pneumonia in children, accounting for 20-33.3% of cases of severe pneumonia (1, 4–6). The fatality rate for untreated severe HAdV-induced pneumonia may exceed 50% (1,4,5), which represents a significant burden on society due to the associated medical expenses (3). Unfortunately, the evaluation of HAdV pneumonia is still based on clinical manifestations and there is a lack of sensitive and specific biomarkers. Furthermore, no adenovirus-specific approved antiviral drugs are currently available for severe cases (7). The early diagnosis and treatment of severe HAdV pneumonia thus remain a major challenge. Therefore, to decrease morbidity and improve prognosis, it is particularly important to explore novel early identification biomarkers and potential antiviral mechanisms in severe cases.

The HAdV infection process is considered to involve RNA splicing and interaction with the innate immunity of the host (8). Notably, HAdV RNA splice sites were detected in a previous study, and some possibly represent noncoding RNAs (9). Nonetheless, the role of noncoding RNA in HAdV pneumonia needs further investigation.

Circular RNAs (circRNAs) have been identified as a class of noncoding RNAs that form as a closed loop from the covalent linkage of the 3' and 5' ends (10). Thus, unlike linear RNA, the special covalently bonded circular structure of circRNA enables high stability and resistance against RNA exonuclease (10). As a novel type of endogenous noncoding RNA, circRNA is expressed widely in different species and has been demonstrated to have tissue specificity (11,12). Due to their stability, abundance, and evolutionary conservation, circRNAs exert key functions in regulating various physiological and pathological processes (13,14). For example, circRNAs can serve as competing endogenous RNAs to inhibit the activity of microRNAs (miRNAs) (10). Increasing evidence indicates that circRNAs play regulatory roles in many human diseases, such as cardiovascular disease, diabetes, and cancer, which make circRNA potential diagnostic and prognostic biomarkers (14-16).

Studies have demonstrated that virus-generated circRNAs or differentially expressed host circRNAs can be used as candidate biomarkers of viral infection (17-19). In addition, circRNAs are involved in regulating the antiviral immunoresponse (17), viral replication (20), and pathogenesis of infectious diseases (19). Moreover, studies have shown that several circRNAs are involved in infectious respiratory diseases (20,21), such as circRNA GATAD2A, which promotes H1N1 replication by inhibiting autophagy (20).

However, there is little information on expression profiling and the potential role of circRNAs in HAdV pneumonia. Therefore, the present study was designed to determine the expression profiles and potential regulatory roles of whole blood circRNA in children with HAdV pneumonia with the aim of identifying a novel diagnostic candidate for HAdV pneumonia in children, particularly severe cases.

## Material and Methods

### Patients and ethics statement

**Figure 1:**
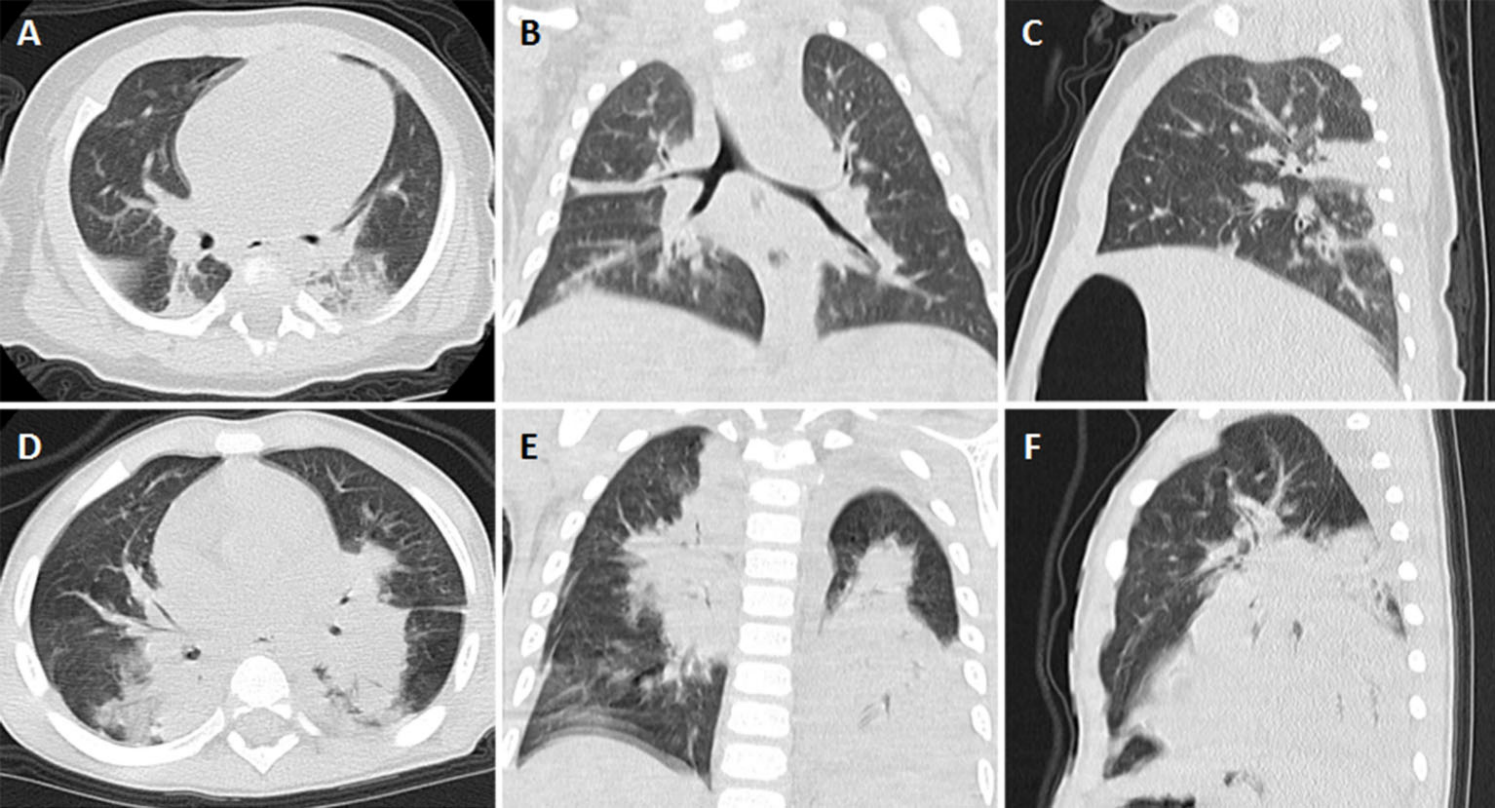
Imaging features of mild and severe human adenoviruses (HAdV) pneumonia patients. **A**-**C**, High-resolution CT scan of the chest at different sections on the day of admission revealing diffuse infiltration and few areas of consolidation in right upper lobe and bilateral lower lobe pneumonia in a three-year-old child with mild HAdV pneumonia; **D**-**F**, High-resolution CT scan of the chest at different sections on the day of admission showing large bilateral consolidation in each lobe and small bilateral pleural effusion in a three-year-old child with severe HAdV pneumonia.

**Figure 2:**
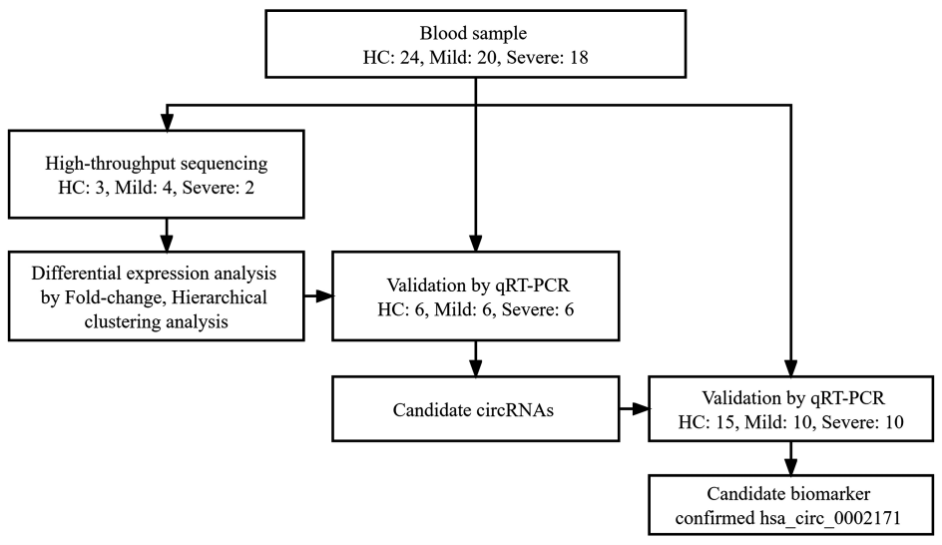
Flowchart of the study. HC: Healthy control group; Mild: Mild human adenoviruses (HAdV) pneumonia group; Severe: Severe HAdV pneumonia group.

Children were recruited from Guangzhou Women and Children's Medical Center. Five microliters of peripheral whole blood was collected using EDTA-anticoagulated BD PAXgene Blood RNA Tubes (BD, USA) from 24 healthy children (denoted as healthy controls), 20 children with mild HAdV pneumonia (denoted as mild), and 18 children with severe HAdV pneumonia (denoted as severe) from March 1, 2019, to August 31, 2019. Samples from 6 of the patients (4 mild and 2 severe) and 3 of the healthy controls were used for high-throughput sequencing, and samples from the other 53 children (21 healthy controls, 16 mild, and 16 severe) were used for validation. Pneumonia was defined as the presence of fever, acute respiratory symptoms (cough, tachypnea, and difficulty breathing), or both, along with the presence of new infiltrates and/or consolidations detected on chest radiography. HAdV infection was identified by positive multiplex polymerase chain reaction (PCR) from nasopharyngeal swabs and/or bronchial alveolar lavage fluid. The exclusion criteria of this study were: 1) evidence of infection with other organisms; 2) corticosteroid use as part of the pre-study therapy; and 3) presence of significant underlying comorbidities, including malnutrition, chronic cardiac or pulmonary disease, tumor, congenital malformation, immunodeficiency, and immunosuppressive medications before admission. Pneumonia severity was classified according to British Thoracic Society guidelines (22). The characteristics of all participants in the study are presented in Table 1. High-resolution chest computed tomography images from children with mild and severe HAdV pneumonia are shown in Figure 1. The flow chart of the present study cohort is shown in Figure 2.

**Table 1:** Characteristics of mild and severe human adenoviruses (HAdV) pneumonia patients for high-throughput sequence.

Clinical information	Healthy (n=24)	Mild (n=20)	Severe (n=18)	P value
Demographic				
Age (months)	20.00 (12.50-35.50)	20.50 (9.75-36.00)	20.00 (11.75-31.00)	0.921^#^
Male gender, n (%)	15 (62.50)	12 (60.00)	10 (55.56)	0.901^#^
Symptoms and signs				
Duration of fever^a^ (days)	N/A	4.5 (4.00-5.00)	8.00 (6.75-11.25)	0.000
Cough, n (%)	N/A	17 (85.00)	18 (100)	0.087
Tachypnea^b^, n (%)	N/A	0 (0)	16 (88.90)	0.000
Laboratory findings^c^				
White blood cell (×10^9^/L)^d^	N/A	7.75 (5.08-11.43)	6.90 (5.30-14.68)	0.919
C-reactive protein (mg/L)^e^	N/A	15.30 (3.75-31.85)	26.22 (5.40-60.21)	0.276
Lactate dehydrogenase (U/L)^f^	N/A	335.00 (292.50-438.75)	610.50 (518.75-1107.75)	0.000
Radiology^g^				
Consolidation, n (%)	N/A	9 (45.00)	16 (88.90)	0.004
Pleural effusion, n (%)	N/A	0 (0)	5 (27.80)	0.011
Management				
Oxygenotherapy, n (%)	N/A	0 (0)	16 (88.90)	0.000
Ventilator support, n (%)	N/A	0 (0)	6 (33.33)	0.005
Duration of hospital stay (days)	N/A	7.00 (6.00-10.75)	14.00 (12.00-18.00)	0.000

Data are reported as number (percentage) or median (25th-75th percentile), where appropriate, and compared with chi-squared test or Mann-Whitney U-test, respectively. For all analyses, 2-tailed P-values were calculated by IBM SPSS Statistics 25.0. Statistical significance was defined as P<0.05. ^#^Compared among healthy control group, mild group, and severe group. ^a^Fever duration from onset to relief. ^b^Respiratory rate>70 breaths/min. ^c^Data extracted from the first test for the children on admission. ^d^The normal reference value was (5-12)×10^9^/L. ^e^The normal reference value was (0-3) mg/L. ^f^The normal reference value was (0-322) U/L. ^g^Judged by chest radiograph or CT scan in whole course of the patients. N/A: not applicable.

The study protocol was conducted in accordance with the Declaration of Helsinki and approved by the Ethics Committee of Guangzhou Women and Children's Medical Center (No. 2017102413). The legal guardians of all participants signed a written informed consent form for the use of the clinical and laboratory data from their medical records.

### RNA isolation

Total RNA was isolated from tissue samples using TRIzol reagent (Life Technologies, USA) according to the manufacturer's instructions. RNA purity was determined using a kaiaoK5500^®^ Spectrophotometer (Kaiao, China). RNA integrity and concentration were assessed using the RNA Nano 6000 Assay kit of the Bioanalyzer 2100 system (Agilent Technologies, USA).

### High-throughput sequencing

A total amount of 3 μg RNA per sample was used as initial material for the RNA sample preparations. Ribosomal RNA was removed using Epicentre Ribo-Zero™ Gold Kits (Human/Mouse/Rat/other) (Epicentre, USA). Subsequently, sequencing libraries were generated by following the manufacturer's recommendations with a varied index label using a NEBNext^®^ Ultra™ Directional RNA Library Prep Kit for Illumina (NEB, USA). Differentially expressed circRNAs and mRNAs were selected with criteria of a P-value <0.05 and log2 fold-change >2 while differentially expressed miRNAs were selected with criteria of a P-value <0.05 and effect size >1.0.

### Quantitative real-time PCR assay

cDNA for circRNA and mRNA was generated using PrimeScript RT reagent Kit (TaKaRa, Japan) according to the manufacturer's protocol. The expressions of circRNA and mRNA were analyzed with a SYBR Premix Ex Taq (TaKaRa) kit. circRNA and mRNA levels were normalized to those of β-actin and evaluated using the 2^−ΔΔCt^ method.

### Data analysis

The R package DESeq2 (v1.26.0; Bioconductor) was used to perform the differential expression analysis in each group using the circRNA and mRNA counts (23). Differentially expressed circRNAs and mRNAs were defined as those with a P-value <0.05 and absolute log2 fold-change >2. These differentially expressed circRNAs were then used to predict the corresponding sponger miRNAs with miRanda (3.3a). The R package multiMiR (v.1.8.0) was used to screen the validated mRNA targets of these miRNAs from databases such as miRecords, miRTarBase, and TarBase (24-26). To investigate the Gene Ontology (GO, http://geneontology.org/) terms associated with the target genes, a hypergeometric P-value was calculated and adjusted as a q-value, with the background set to genes in the whole genome. GO terms with q<0.05 were considered to be significantly enriched. GO enrichment analysis identifies the biological functions of the DEGs. KEGG (Kyoto Encyclopedia of Genes and Genomes, http://www.kegg.jp/) is a database resource containing a collection of manually drawn pathway maps representing our knowledge of the molecular interaction and reaction networks. Using the same method as GO enrichment analysis, significantly enriched KEGG pathways were identified. GO terms and KEGG pathways with P-values <0.05 were considered significantly enriched.

### Statistical analysis

All data are reported as means±SD. Comparisons between the healthy control and HAdV pneumonia groups and between the mild and severe HAdV pneumonia groups were performed using a two-tailed Student's *t*-test. Differences were considered significant at P<0.05.

## Results

### Identification of differentially expressed circRNA profiles in children with HAdV pneumonia

**Figure 3:**
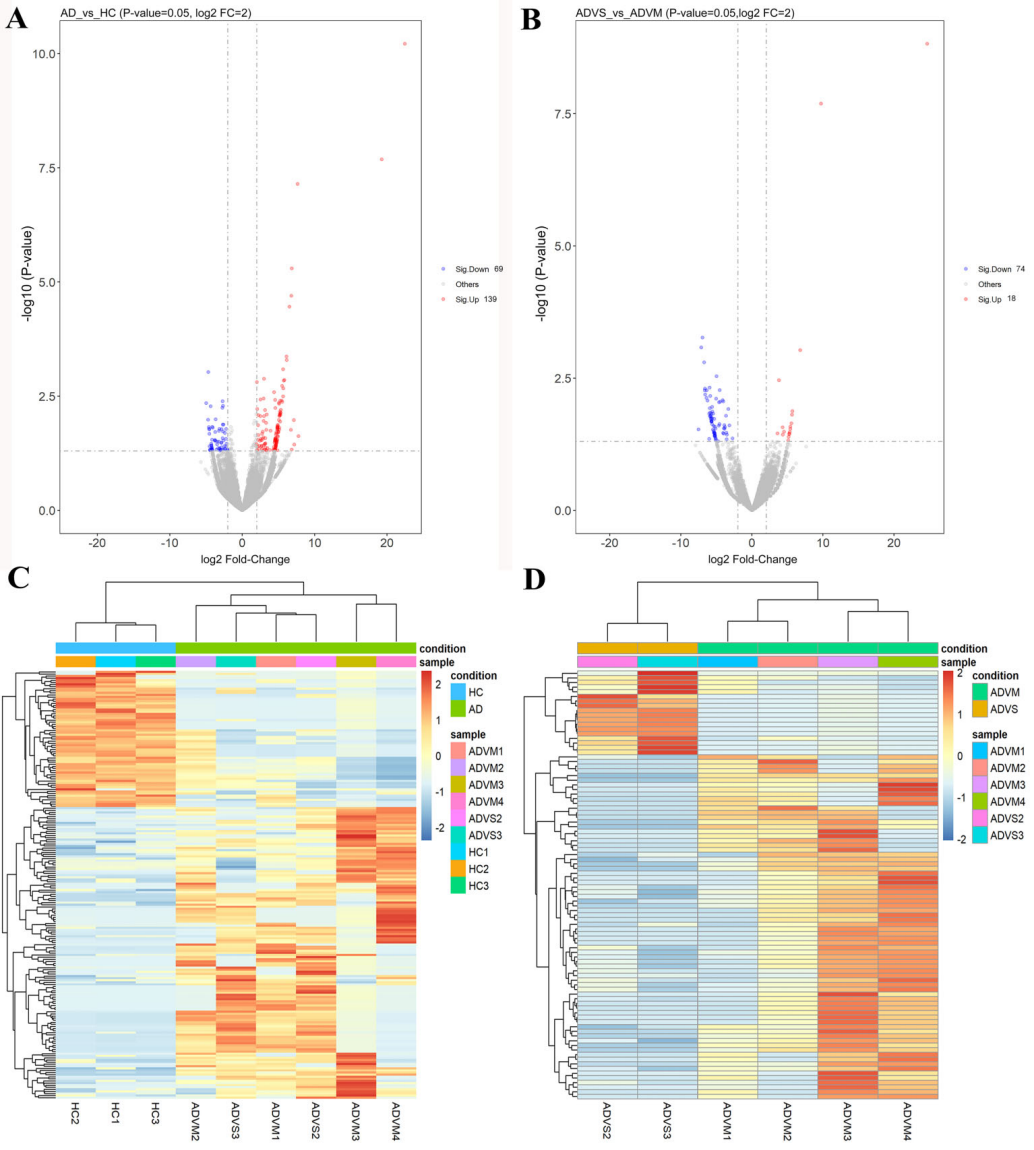
The circRNAs expression profile of whole blood from children with human adenoviruses (HAdV) pneumonia. Volcano plot (**A**) and hierarchical cluster analysis (**C**) of differentially expressed circRNAs between HAdV pneumonia group and healthy control group. Volcano plot (**B**) and hierarchical cluster analysis (**D**) of differentially expressed circRNAs between mild HAdV pneumonia group and severe HAdV pneumonia group.

To explore the differentially expressed circRNAs in HAdV pneumonia patients, we collected whole blood samples from three healthy children, four children with mild HAdV pneumonia, and two children with severe HAdV pneumonia for circRNA profiling. By comparing samples between children with HAdV pneumonia and healthy controls, we were able to identify the roles of circRNAs in HAdV pneumonia, while by comparing samples between the mild and severe groups, we were able to analyze the role of circRNAs in severe pediatric HAdV pneumonia. We identified 208 circRNAs that were differentially expressed in samples from children with HAdV pneumonia *vs* samples from healthy controls (Figure 3A), while 92 circRNAs were differentially expressed in samples from the severe group *vs* samples from the mild group (Figure 3B). Hierarchical cluster analysis showed that our fold-change filter (log2 fold-change >2.0 and P<0.05) was passed by 69 upregulated circRNAs plus 139 downregulated circRNAs in the samples from HAdV-infected children (Figure 3A and C) and by 74 upregulated circRNAs plus 18 downregulated circRNAs in the samples from the severe group (Figure 3B and D). The top 10 up- and downregulated circRNAs between children with HAdV pneumonia and healthy controls are listed in Table 2. The top 10 up- and downregulated circRNAs between the mild and severe groups are shown in Table 3. These data indicated that the expression of circRNAs differed between children with HAdV pneumonia and healthy controls and between children with severe HAdV pneumonia and children with mild HAdV pneumonia.

**Table 2:** The top 20 differentially expressed circRNAs ranked by human adenoviruses pneumonia (HP) *vs* healthy control (HC) group.

circRNA	Base mean	log2 fold-change	HP *vs* HC	P-value
hsa_circ_0002171	45.11659	22.4719	Upregulation	6.09E-11
hsa_circ_0022787	20.04478	19.267	Upregulation	2.06E-08
hsa_circ_0001535	16.6864	7.76926	Upregulation	2.38E-02
hsa_circ_0027653	15.50299	7.64992	Upregulation	7.17E-08
hsa_circ_0014890	11.33416	7.19782	Upregulation	0.036312
hsa_circ_0005013	131.4455	7.12015	Upregulation	1.06E-02
hsa_circ_0027651	8.873884	6.85453	Upregulation	5.06E-06
hsa_circ_0025965	8.81343	6.84246	Upregulation	4.67E-02
hsa_circ_0027659	8.203528	6.77815	Upregulation	2.01E-05
hsa_circ_0014467	8.333656	6.73995	Upregulation	0.017348
hsa_circ_0001342	2.335308	4.964873	Downregulation	0.004494
hsa_circ_0009475	1.96929	4.719592	Downregulation	0.010462
hsa_circ_0007574	5.376817	4.680545	Downregulation	0.00094
hsa_circ_0022594	1.82025	4.606534	Downregulation	0.015071
hsa_circ_0005988	1.771918	4.567081	Downregulation	0.016403
hsa_circ_0004331	1.751385	4.546269	Downregulation	0.021264
hsa_circ_0011797	1.745957	4.544233	Downregulation	0.017025
hsa_circ_0008908	1.715948	4.520099	Downregulation	0.039414
hsa_circ_0013358	1.640501	4.451646	Downregulation	0.047122
hsa_circ_0009250	1.629224	4.441936	Downregulation	0.047974

**Table 3:** The top 20 differentially expressed circRNAs ranked by mild human adenoviruses (HAdV) pneumonia (Mild) *vs* severe HAdV pneumonia (Severe) group.

circRNA	Base mean	log2 fold-change	Mild *vs* Severe	P-value
hsa_circ_0014349	142.1792	24.6603	Upregulation	1.51E-09
hsa_circ_0002171	56.4688	9.72026	Upregulation	2.05E-08
hsa_circ_0003646	7.449894	6.79788	Upregulation	0.000938
hsa_circ_0024342	3.496868	5.70687	Upregulation	0.013308
hsa_circ_0009627	3.342354	5.64191	Upregulation	0.01563
hsa_circ_0018396	3.118229	5.54121	Upregulation	0.022713
hsa_circ_0021791	2.846116	5.40948	Upregulation	0.029148
hsa_circ_0001126	2.820276	5.39669	Upregulation	0.026219
hsa_circ_0018418	2.66207	5.31379	Upregulation	0.033422
hsa_circ_0020848	2.555545	5.25442	Upregulation	0.035095
hsa_circ_0024426	18.27594	7.524762	Downregulation	0.02967
hsa_circ_0000198	14.10296	7.143591	Downregulation	0.000836
hsa_circ_0010310	12.53535	6.975257	Downregulation	0.00054
hsa_circ_0012805	10.72797	6.732803	Downregulation	0.001595
hsa_circ_0010461	9.678138	6.627842	Downregulation	0.005383
hsa_circ_0002175	9.703208	6.597645	Downregulation	0.00503
hsa_circ_0003405	9.770264	6.590154	Downregulation	0.006486
hsa_circ_0007598	8.516139	6.412464	Downregulation	0.007532
hsa_circ_0004205	8.258286	6.379966	Downregulation	0.005395
hsa_circ_0006640	7.739889	6.260617	Downregulation	0.008475

### Validation of the differentially expressed circRNAs using qRT-PCR

**Figure 4:**
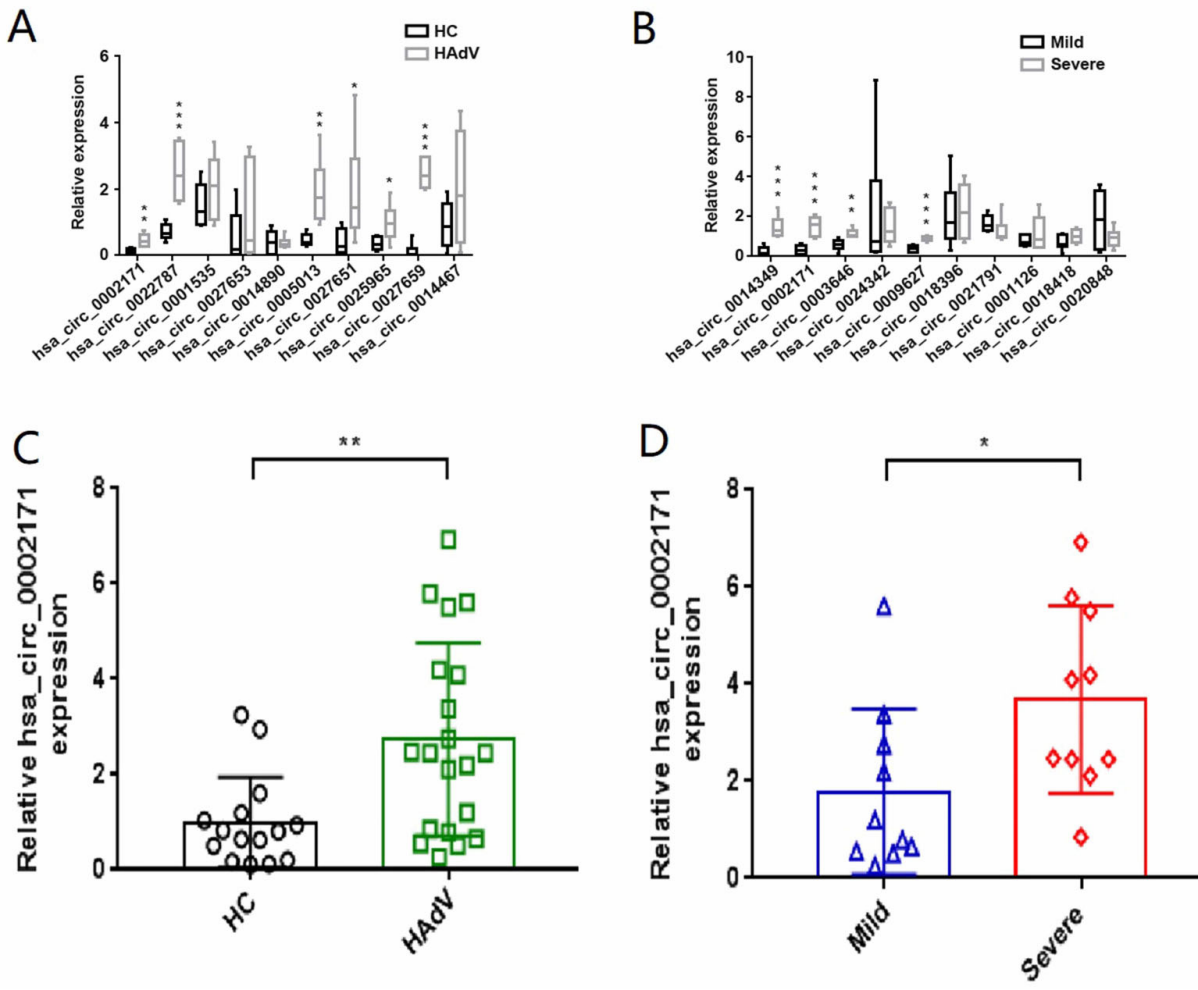
Quantitative validation of the differentially expressed circRNAs expression levels by qRT-PCR. Quantification of top 10 upregulated expressed circRNAs between human adenoviruses (HAdV) pneumonia group (n=6) and healthy control (HC) group (n=6) (**A**) and between mild HAdV pneumonia group (n=6) and severe HAdV pneumonia group (n=6) (**B**). Quantification of differentially expressed circRNAs (hsa_circ_0002171) among both children with HAdV pneumonia (n=20) *vs* healthy control group (n=15) (**C**) and mild HAdV pneumonia group (n=10) *vs* severe HAdV pneumonia group (n=10) (**D**). Data are reported as means±SD from independent experiments. *P<0.05, **P<0.01, and ***P<0.001 (Student’s *t*-test).

To confirm the high-throughput sequence data, we selected the differentially expressed circRNAs in Table 2 and Table 3 for further validation with quantitative real-time PCR (qRT-PCR) using six samples from each group. We found that hsa_circ_0002171, hsa_circ_0022787, hsa_circ_0005013, hsa_circ_0027651, hsa_circ_0025965, and hsa_circ_0027659 were upregulated in samples from children with HAdV pneumonia *vs* healthy children (Figure 4A) while hsa_circ_0014349, hsa_circ_0002171, hsa_circ_0003646, and hsa_circ_0009627 were upregulated in samples from the severe group *vs* the mild group (Figure 4B), which was consistent with our sequencing data. In particular, we found that hsa_circ_0002171 was differentially expressed between children with HAdV pneumonia and healthy control children and between the severe and mild groups. Based on the qRT-PCR results of 10 samples from the severe group, 10 samples from the mild group, and 15 samples from the healthy control group, we found that hsa_circ_0002171 was significantly elevated in samples from children with HAdV pneumonia compared with healthy controls (Figure 4C) and in samples from the severe group compared with the mild group (Figure 4D). Thus, hsa_circ_0002171 can likely be considered a candidate biomarker for both HAdV pneumonia and severe HAdV pneumonia in children.

### Transcriptional regulation of circRNAs in children with HAdV pneumonia

**Figure 5:**
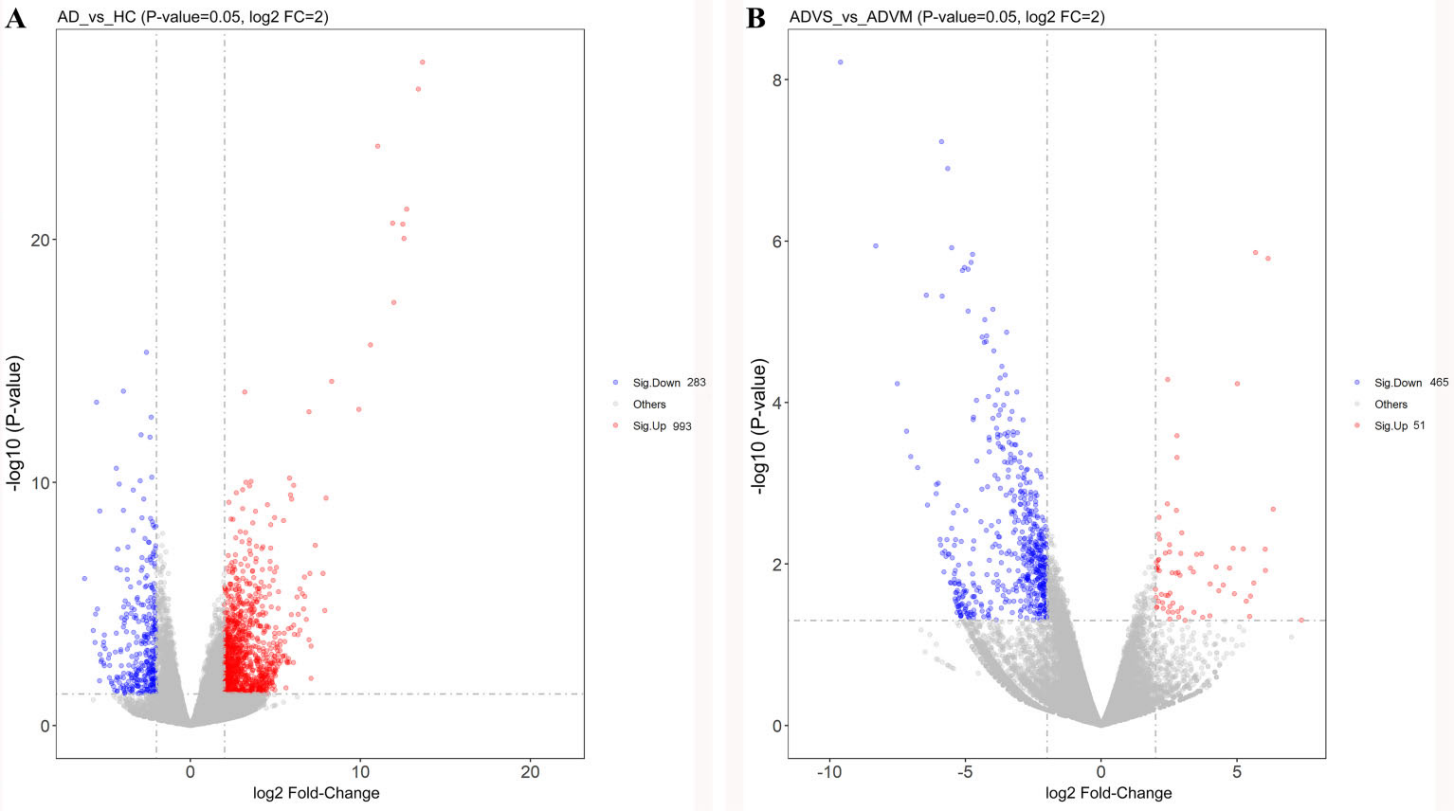
RNA-sequencing of differentially expressed mRNAs. Volcano plot of differentially expressed target mRNAs between human adenoviruses (HAdV) pneumonia group and healthy control group (**A**). Volcano plot of differentially expressed target mRNAs between mild HAdV pneumonia group and severe HAdV pneumonia group (**B**).

**Figure 6:**
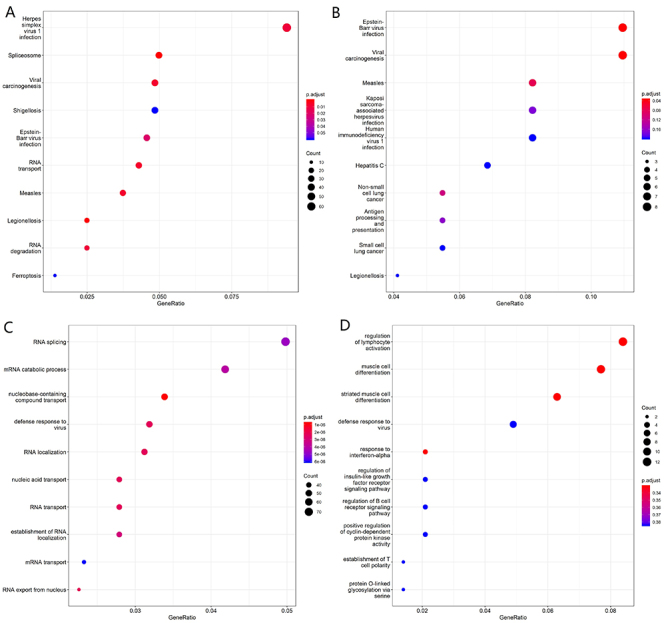
KEGG and Gene Ontology (GO) enrichment analysis of the differentially expressed target genes. The KEGG pathway scatterplot of target genes between healthy control group and human adenoviruses (HAdV) pneumonia group (**A**) and between mild and severe HAdV groups (**B**). The GO pathway scatterplot of target genes between healthy control group and HAdV pneumonia group (**C**) and between mild and severe HAdV groups (**D**).

**Figure 7:**
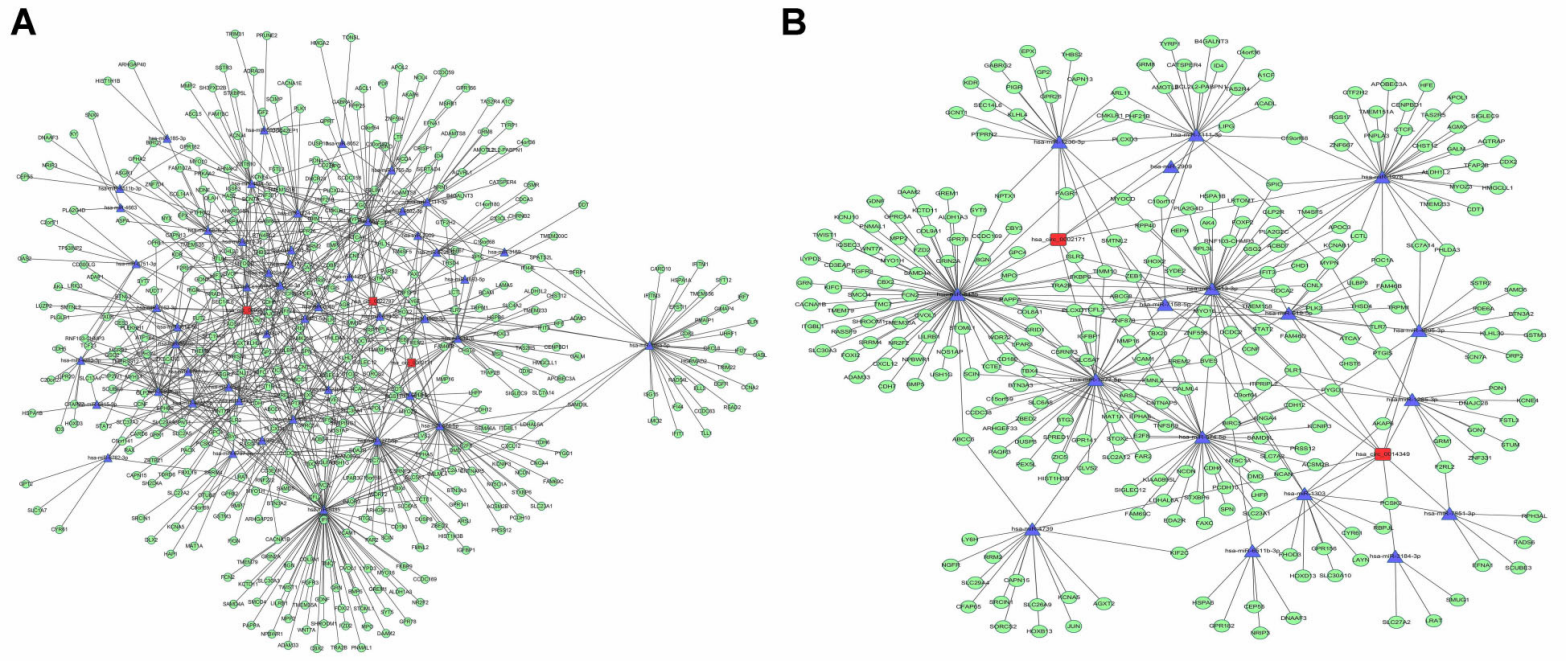
The circRNA-miRNA-mRNA regulatory network in human adenoviruses (HAdV) pneumonia group. The circRNA-miRNA-mRNA regulatory networks from circRNA-mRNA gene pairs between HAdV pneumonia group *vs* healthy control group (**A**) and between severe group *vs* mild group (**B**) was analyzed using Cytoscape software. Squares, triangles, and circles represent circRNA, miRNA, and target genes, respectively. Orange nodes represent genes that are upregulated in severe HAdV pneumonia, and blue nodes represent downregulated genes.

To further explore the possible mechanisms of the differentially expressed circRNAs in children with HAdV pneumonia, we performed RNA sequencing and constructed the circRNA-mRNA regulatory network. We combined the differentially expressed mRNAs and circRNAs with circRNA target prediction to obtain genuine circRNA targets in children with HAdV pneumonia. According to the results of the differentially expressed mRNA and circRNA expression analysis, the regulatory relationships between circRNAs and mRNAs were predicted. Based on the results, our fold-change filter (log2 fold-change >2.0 and P<0.05) was passed by 283 upregulated target mRNAs plus 993 downregulated target mRNAs in the samples from children with HAdV pneumonia compared with healthy control children (Figure 5A) and by 465 upregulated target mRNAs plus 51 downregulated target mRNAs in the samples from the severe group compared with the mild group (Figure 5B). We further performed functional enrichment analysis of differentially expressed mRNAs, including KEGG pathway analysis (Figure 6A and B) and GO analysis (Figure 6C and D). The top 10 enriched GO terms and pathways of differentially expressed mRNAs were ranked by enrichment score. KEGG pathway analysis of the differentially expressed mRNAs between healthy controls and children with HAdV pneumonia revealed that the differentially expressed mRNAs were involved in viral infection (Figure 6A), while that of the differentially expressed mRNAs between the mild and severe groups revealed that these differentially expressed mRNAs were also involved in viral infection (Figure 6B). However, GO analysis of the differentially expressed mRNAs between healthy controls and children with HAdV pneumonia indicated “RNA processing pathway” (Figure 6C) while GO analysis of the differentially expressed mRNAs between the mild and severe groups indicated “immune pathway” (Figure 6D). The differentially expressed mRNAs of the top 1 enriched GO pathway are shown in Table 4. We further established the circRNA-miRNA-mRNA gene regulatory networks from the above circRNA-mRNA gene pairs between the HAdV pneumonia and healthy control groups (Figure 7A) and the severe and mild groups (Figure 7B) using Cytoscape software. These results indicated that the genes involved in mild HAdV pneumonia differed from the genes involved in severe HAdV pneumonia. These genes may reflect the mechanisms of HAdV infection and HAdV-induced pneumonia.

**Table 4:** The differentially expressed mRNAs of the top 1 enriched Gene Ontology pathway.

Group	Top 1 pathway	mRNAs
HC *vs* HP	RNA splicing	*HSPA1A*, *LSM4*, *PPWD1*, *PIK3R1*, *FUS*, *SRSF3*, *COIL*, *KHDC4*, *SMU1*, *SRSF7*, *RBM28*, *CLK4*, *PRPF40A*, *HNRNPA3*, *SON*, *FASTK*, *SRSF2*, *SRSF10*, *KDM1A*, *DDX39B*, *U2SURP*, *PPP2CA*, *TARDBP*, *SYF2*, *HNRNPF*, *SRRM1*, *PTBP2*, *SRSF11*, *EIF4A3*, *HNRNPA2B1*, *DCPS*, *SLU7*, *CWF19L1*, *C2orf49*, *PCBP2*, *PPIL1*, *SRSF5*, *RBM8A*, *ZCCHC8*, *DHX40*, *CPSF2*, *BCAS2*, *YTHDC1*, *SNW1*, *HNRNPD*, *TRA2B*, *SF3B1*, *PRPF4*, *HNRNPA1*, *HNRNPA1L2*, *SREK1*, *WTAP*, *ZNF326*, *PRPF4B*, *DDX5*, *SMNDC1*, *METTL16*, *GEMIN6*, *HSPA8*, *PHF5A*, *NUP98*, *RBMXL1*, *HNRNPC*, *CLK1*, *HNRNPL*, *SRSF6*, *SF1*, *PPIG*, *SNRNP40*, *THOC1*, *PRPF8*, *RPS26*, *DHX35*, *PNN*, *PABPC1*
Mild *vs* Severe	Regulation of lymphocyte activation	*CDKN1A*, *ZFP36L1*, *DTX1*, *FANCA*, *SDC4*, *RARA*, *ICOSLG*, *TNFRSF13C*, *GSN*, *CD19*, *NFAM1*, *FLOT2*

HC: Healthy control group; HP: human adenoviruses pneumonia (HAdVs) group; Mild: mild HAdVs pneumonia group; Severe: severe HAdVs pneumonia group.

### ROC curve analysis of confirmed circRNAs

**Figure 8:**
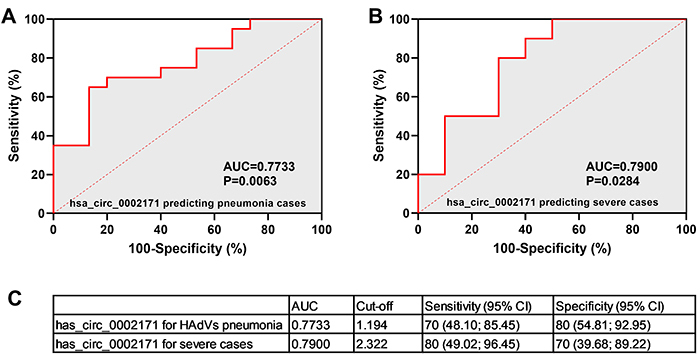
Hsa_circ_0002171 may be a potential indicator of human adenoviruses (HAdV) pneumonia and severe cases. Receiver operating characteristic (ROC) analysis of the ability of hsa_circ_0002171 to predict HAdV pneumonia (**A**) and severe cases (**B**). Cut-off points of hsa_circ_0002171 as well as their sensitivity and specificity for prediction of HAdV pneumonia and severe cases (**C**).

Receiver operating characteristics (ROC) curve analysis was conducted to evaluate the potential diagnostic value of significant differentially expressed circRNAs. ROC curves of confirmed circRNAs showed that the level of hsa_circ_0002171 could separate patients with HAdV pneumonia from healthy controls and severe cases from mild cases. The results of ROC curve analysis indicated diagnostic accuracies of hsa_circ_0002171 in predicting HAdV pneumonia and severe cases of 77.33 and 79.00%, respectively (Figure 8A and B). If the cutoff value of hsa_circ_0002171 was set at 0.8401, the sensitivity and specificity for predicting HAdV pneumonia were 70 and 80%, respectively, while they were 80 and 70%, respectively, for predicting severe HAdV pneumonia cases at the cutoff value of 1.633 (Figure 8C). Thus, it appears that hsa_circ_0002171 may be valuable as a biomarker for HAdV pneumonia diagnosis.

## Discussion

HAdVs play a significant role in pediatric pneumonia and are frequently associated with severe pediatric pneumonia, causing significant morbidity and mortality in infants and children (27). However, although high mortality rates are often reported in cases of severe HAdV pneumonia, there is no specific intervention for severe HAdV pneumonia and the underlying mechanisms of the pneumonia remain to be determined (6). Severe HAdV pneumonia may cause severe long-term sequelae or even death (1). Thus, investigation of the molecular network of interactions between HAdVs and host factors and elucidation of the biomarkers of severe HAdV pneumonia are crucial to the early diagnosis of the disease and the identification of innovative therapeutic targets.

The severity of pediatric HAdV pneumonia is associated with the host's age and immune state and the HAdV subtype (5,6). Current recognition of severe HAdV pneumonia primarily relies on the clinical characteristics manifesting in the late stage (6). There is thus still a lack of indicators for the early specific recognition of HAdV pneumonia, especially severe cases.

circRNAs exert ubiquitous biological functions in various tissues and cells (13,14,28). They have recently been reported as a unique class of long noncoding RNAs that affect the expression of a target miRNA and thereby regulate mRNA expression and influence many biological processes (29,30). circRNAs have been identified as novel diagnostic and therapeutic biomarkers for several diseases (14,31). Noncoding RNAs are considered to be involved in the RNA splicing associated with HAdV infection (9). It was reported that highly upregulated miRNAs may play crucial roles in HAdV pneumonia pathogenesis and are potential biomarkers (32). Moreover, studies suggested that several circRNAs are involved in H1N1 replication (20,21). However, no studies have reported the expression profiles and roles of circRNAs in whole blood from children with HAdV pneumonia. Hence, it would be meaningful to profile circRNA expression and search for new biomarkers, which may help to provide new directions and strategies for disease diagnosis.

In the present study, circRNA expression profiles in whole blood from children with HAdV pneumonia were first analyzed and validated. In total, 208 differentially expressed circRNAs were found in whole blood from HAdV pneumonia *vs* healthy children, including 69 upregulated and 139 downregulated circRNAs. In addition, 92 differentially expressed circRNAs, including 74 upregulated and 18 downregulated circRNAs, were identified in children with mild *vs* severe HAdV pneumonia. Through further qRT-PCR validation experiments with an increased sample size, 6 and 4 of the top 10 upregulated circRNAs were identified between the HAdV pneumonia and healthy control groups and between the severe and mild HAdV pneumonia groups, respectively. These findings suggested that HAdV pneumonia can alter circRNA expression patterns in children and that this change may be related to the severity of the disease. The results also suggested that differentially expressed circRNAs may be involved in regulating the pediatric HAdV pneumonia process. Our results may enrich the study of the pathogenesis of HAdV pneumonia and provide a theoretical basis for the in-depth exploration of the function of circRNAs in HAdV pneumonia, particularly in severe cases. These differentially expressed circRNAs might be involved in HAdV pneumonia, although the exact mechanism requires further investigation.

In this study, we found that a set of circRNAs might be useful as biomarkers for pediatric HAdV pneumonia. Previous work has shown that noncoding RNAs in whole blood can be used as potential biomarkers, such as hsa-miR-127-3p, hsa-miR-493-5p, and hsa-miR-409-3p (32). However, no association between these markers and disease severity has been established, and their sensitivity and specificity are unclear. In our study, the differential expression of hsa_circ_0002171 was detected between the HAdV pneumonia and healthy control groups, as well as between the severe and mild groups. As expected, the average expression levels of hsa_circ_0002171 were significantly higher in the whole blood of patients with HAdV pneumonia than in that of healthy controls. Furthermore, the expression of hsa_circ_0002171 was even higher in the whole blood of severe cases than in that of mild cases. Accordingly, we found that the expression levels of hsa_circ_0002171 were positively correlated with the severity of HAdV pneumonia in children. Through ROC curve analysis, hsa_circ_0002171 showed significant utility in the diagnosis of HAdV pneumonia and of severe cases, with area under the curve (AUCs) of 0.7733 (95%CI: 0.6183-0.9284, P=0.0063) and 0.7900 (95%CI: 0.5861-0.9939, P=0.0284), respectively. Its diagnostic value for severe cases was higher than that of procalcitonin, interleukin 6, the erythrocyte sedimentation rate, C-reactive protein, and prealbumin, which all had AUCs less than 0.656 in previous research (33). Our results thus indicated that hsa_circ_0002171 may have potential as a novel biomarker for the diagnosis of pediatric HAdV pneumonia and prediction of severe cases, also proving that hsa_circ_0002171 is of considerable importance for practical applications. Unfortunately, to the best of our knowledge, the biological role of hsa_circ_0002171 has not yet been reported.

After integrating the transcriptome analysis, we identified the target mRNAs of the differentially expressed circRNAs. Through GO analysis of the differentially expressed target mRNAs, we found that the differentially expressed genes between healthy controls and HAdV pneumonia patients were mainly involved in RNA splicing. This is consistent with the presence of RNA splicing during adenovirus infection (8). Several genes have been reported to be involved in viral replication, including *FUS*, *PPP2CA*, *EIF4A3*, *PCBP2*, and *TARDBP*, which are implicated in the mechanism underlying HAdV pneumonia. In contrast, when we analyzed the GO enrichment of differentially expressed genes between children with mild and severe HAdV pneumonia, we found that the genes were mainly involved in lymphocyte activation. These results indicated that severe HAdV pneumonia is not only associated with virus infection, but also involved in the triggering of an excessive host immune response during infection. Thus, the treatment for severe HAdV pneumonia should be combined with immunotherapy. This is consistent with the greater need for immunomodulatory therapy (such as glucocorticoids and intravenous immunoglobulin) in the treatment of severe HAdV pneumonia (6). The differentially expressed genes between children with mild and severe HAdV-induced pneumonia may reflect differences in the pathogenic mechanisms.

In summary, we identified an altered profile of circRNA expression in whole blood from children with HAdV pneumonia. The circRNA hsa_circ_0002171 plays a potential role in the diagnosis and disease severity prediction of HAdV pneumonia. Simultaneously, based on the transcriptional regulation of circRNAs, we found that genes related to RNA splicing and the regulation of lymphocyte activation may play important roles in HAdV pneumonia. However, considering the small sample size of this study, further studies are needed to clarify the source, role, and mechanism in these processes, which will provide a physiological basis for the diagnosis of pediatric HAdV pneumonia.
